# Simple clinical risk score for no-reflow prediction in patients undergoing primary Percutaneous Coronary Intervention with acute STEMI

**DOI:** 10.12669/pjms.313.7484

**Published:** 2015

**Authors:** Nazile Bilgin Dogan, Ebru Ozpelit, Selma Akdeniz, Muzaffer Bilgin, Nezihi Baris

**Affiliations:** 1 Nazile Bilgin Dogan, MD. Department of Cardiology, Menemen State Hospital, Izmir, Turkey; 2 Ebru Ozpelit, MD. Department of Cardiology, DokuzEylul University Hospital, Izmir, Turkey; 3 Selma Akdeniz, MD. Department of Cardiology, AKUT Cardiovascular Hospital, Izmir, Turkey; 4 Muzaffer Bilgin, M.Sc. Department of Biostatistics, Eskisehir Osmangazi University, Eskisehir, Turkey; 5 Prof. Dr. Nezihi Baris, Professor, Department of Cardiology, DokuzEylul University Hospital, Izmir, Turkey

**Keywords:** No-reflow, Risk score, ST-segment elevation myocardial infarction (STEMI), Percutaneous coronary intervention

## Abstract

**Objectives:**

To identify the STEMI patients at high risk in terms of no-reflow during percutaneous coronary intervention (PCI) with a simple risk score system that can be used before reperfusion.

**Methods:**

Total 173 patients who had undergone primary or rescue percutaneous coronary intervention following the diagnosis of STEMI, were classified as “no-reflow” developers and “no-reflow” non-developers, during the procedure. The pre-procedural ECGs, laboratory parameters, demographic data, time for the treatment, and the treatment methods were evaluated with univariate analysis. The independent predictors were identified by multivariate logistic regression analysis among the no-reflow risk factors. Using the independent predictors, we developed a simple risk score system proportional to area under the ROC (AUROC) curves.

**Results:**

The independent predictors of “no-reflow” phenomenon were identified as follows: high values of blood glucose at reference; long symptom-onset-to-balloon-time; and low lymphocyte count. The incidence rates of “no-reflow” in patients with low (0-1), moderate (2-3) and high (4-6) risk factors were 13.3%, 40.0%, and 46.7%, respectively. The risk score system demonstrated a good risk prediction between patients with various risk levels of the development of “no-reflow” with a c-statistics of 0.734 (95% CI 0.654-0.814).

**Conclusion:**

The development of “no-reflow” which is an adverse event in STEMI treatment can be predicted efficiently by simple clinical risk scoring method.

## INTRODUCTION

Acute coronary syndromes including the ST elevation myocardial infarction (STEMI) are the most important conditions of the ischemic heart diseases. Increase in invasive interventions in acute coronary syndromes has resulted in newer complications. In percutaneous coronary intervention; the phenomenon of no-reflow is defined as inadequate myocardial perfusion through a given segment of the coronary circulation without angiographic evidence of mechanical vessel obstruction. Dangerous arrhythmias, congestive heart failure, and cardiac death are seen more frequently in patients who develop “no-reflow” after AMI.

The choice of appropriate treatment by the identification of risk predictors in the pathophysiology of “no-reflow” phenomenon would significantly improve the prognosis of the procedure. Hence, in this study, it was aimed to identify the high risk patients before the procedure on the basis of the evaluation of demographics, laboratory parameters, ECGs, symptom-balloon time and the treatment methods of the patients that underwent primary or rescue percutaneous coronary intervention with the diagnosis of STEMI.

## METHODS

### Patients

One hundred seventy three patients older than 18 years who were hospitalized and had undergone primary or rescue PCI at DokuzEylül University Medical Faculty Coronary Intensive Care Unit between January 2009 and August 2011, with the diagnosis of STEMI were included in the study. STEMI was diagnosed with the presence of chest pain with electrocardiographic changes (ST-segment elevation of >1 mm in at least two extremity electrocardiographic leads or 2 mm in at least two consecutive precordial leads). Exclusion criteria were as follows: performed percutaneous interventions for stable angina pectoris or unstable angina pectoris or non-ST elevation myocardial infarction (NSTEMI), patients with malignancies, coagulation disorders. Detailed demographic, clinical, electrocardiographic, angiographic, and procedural data were collected from patient records. The study protocol was approved by the Ethical Committee of the University (Ethics Committee approval number: 2011/18-10).

### Laboratory tests and revascularization procedure

The study was retrospective and all data were acquired from patient history files. Blood samples for complete blood count, glucose, renal functions, and cardiac biomarkers of STEMI patients were drawn at admission to the emergency service in DokuzEylul University. Hemoglobin A1c and lipid parameters were measured after 24 hours from admission to emergency service at the intensive care unit. The end procedural angiographic TIMI flow which is reported in patient’s catheter report was used for the diagnosis of “no-reflow”. The end procedural TIMI grades 0, 1, and 2 flows were described as “no-reflow” phenomenon. In DokuzEylul University Hospital, acetylsalicylic acid (ASA), clopidogrel therapy and intravenous heparin before PCI were administered as a standard therapy at catheterization room. GP IIb/IIIa inhibitors and/or aspiration catheter were preferred according to the coronary angiography findings. Treatment with balloon plus stent or direct stent was determined by the operator according to the characteristics of the lesion.

### Statistical analysis

Statistical analysis was performed using SPSS v15.0 (Statistical Package for Social Sciences) and MedCalc 13.1.2. The continuous variables were presented as mean (±SD) and categorical variables as percentages (n(%)). Normal distributed variables were assessed with independent t-test, not normal distributed variables were assessed with Mann-Whitney U test, and all the variables were compared between both the groups. The adequacy of data for normal distribution was tested by Shapiro-Wilk test. Categorical data were compared with chi-square test. One sample z (test and confidence interval) was used for testing ratios. Univariate analysis and multivariate logistic regression models were used to identify the risk factors of no-reflow before PCI. Receiver-operating characteristics (ROC) curve analysis of categorical variables was performed to identify the optimal cutoff value for predicting no-reflow phenomenon. The p value <0.05 was considered to indicate statistical significance.

## RESULTS

A total of 173 patients (33 females (19.1%) and 140 males (80.9%)) with a mean age of 58.43±11.46 years (min: 33.0, max: 85.0) were included in the study. Risk factors for the development of “no-reflow” were evaluated individually with reference to demographic characteristics, laboratory parameters, ECGs, pre-procedural medicines, initiation of symptoms, treatment, therapies given during the PCI, and STEMI treatment methods (Table-I). When all of the significant parameters were evaluated with multivariate logistic regression analysis, the independent predictors of “no-reflow” phenomenon were found as follows: high blood glucose levels at admission (OR = 1.008; 95% CI = 1.002, 1.013; p = 0.004), long symptom-onset-to-balloon-time (OR = 1.486; 95% CI = 1.248, 1.770; p <0.001) and low lymphocyte count (OR = 0.999; 95% CI = 0.999, 1.000; p = 0.026) (Table-II).

**Table-I:** Study Population Basic Characteristics.

Variable	No-reflow (n:45, %26)	Normal reflow (n:128, %74)	P value
Demographic characteristics			
Age (mean ± SD*)	61.33±12.34	57.41±11.01	0.048
Women (n**, %)	16 (35.6)	17 (13.3)	0.002
Prior Stent (n**, %)	6 (87.5)	19 (90.5)	0.724
Prior CABG (n**, %)	1 (14.3)	2 (9.5)	
Hypertension (n**, %)	23 (51.1)	52 (40.6)	0.296
Diabetes mellitus (n**, %)	13 (28.9)	16 (12.6)	0.023
Smoking status (n**, %)	25 (56.8)	93(72.7)	0.078
Current smoker (n**, %)	18 (75)	74 (84.1)	0.367
Previous smoker (n**, %)	6 (25)	14 (15.9)	
Smoking duration (mean ± SD*)	40.00±12.24	30.875±10.49	0.085
*Medication usage before MI*			
ASA (n**, %)	6 (13.3)	24 (18.8)	0.55
Klopidogrel *** (n**, %)	2 (4.4)	1 (0.8)	
Statin (n**, %)	6 (13.3)	12 (9.4)	0.57
Insulin (n**, %)	3 (25)	4 (26.7)	1.000
OAD (n**, %)	8 (66.7)	10 (66.7)	
OAD+insulin (n**, %)	1 (8.3)	1 (6.7)
Laboratory parameters on admission			
Blood glucose level (mg/dl) (mean ± SD*)	208.18±133.9	147.01±49.7	0.014
Hb (g/dl) (mean ± SD*)	13.19±2.2	14.26±1.9	0.005
MPV (mean ± SD*)	8.39±1.0	8.40±1.0	0.954
Neutrophil count (mean ± SD*)	9323.0±4815.8	8983.76±3752.7	0.631
Lymphocyte count (mean ± SD*)	1920.91±1595.0	2592.88±1922.3	0.023
Neutrophil/lymphocyte ratio (mean ± SD*)	8.61±9.9	5.88±5.0	0.086
WBC count (mean ± SD*)	12204.44±4997.4	12037.91±3969.6	0.822
PLT count (mean ± SD*)	231822.2±77262.6	239152.1±70260.7	0.559
Sedm (mean ± SD*)	18.40±12.8	18.21±15.2	0.956
CRP (mean ± SD*)	18.52±37.0	11.14±24.4	0.213
BNP(pg/ml) (mean ± SD*)	277.28±469.0	274.17±393.0	0.617
LDL (mg/dl) (mean ± SD*)	117.46±35.0	120.34±40.3	0.689
HDL(mg/dl) (mean ± SD*)	35.74±7.7	36.55±9.0	0.614
T. cholesterol (mg/dl)(mean ± SD*)	185.10±46.7	184.45±39.1	0.931
Triglycerides (mg/dl)(mean ± SD*)	159.28±97.8	149.73±98.6	0.286
Creatinine (mean ± SD*)	1.06±0.4	0.94±0.3	0.229
HbA1c (mean ± SD*)	6.55±1.9	6.59±1.7	0.442
ECG characteristics and treatment duration			
Mean ST elevation (mm) (mean ± SD*)	10.95±5.1	9.48±5.9	0.048
Mean ST depression (mm) (mean ± SD*)	5.82±4.94	5.04±4.91	0.136
QT duration (msn) (mean ± SD*)	369.50±39.4	354.09±33.98	0.023
Heart rate (beat/min) (mean ± SD*)	83.07±20.80	78.34±17.69	0.166
Anterior MI (n**, %)	20 (%44.4)	61 (%47.7)	0.901
Inferior MI (n**, %)	24(%53.3)	62 (%48.4)	
Lateral MI (n**, %)	1(%2.2)	5 (%3.9)	
Symptom-ballon time (h)	5.79±4.5	2.43±2.2	0.000
Treatment during procedure			
Existence of thrombus	19 (43,2)	59 (46,1)	0,874
Glycoprotein IIb/IIIa inhibitor	29 (64,4)	65 (50,8)	0,159
Aspiration catheter (aspiration thrombectomy)	16 (36,4)	41 (32,0)	0,733
Direct stenting	7 (15,6)	44 (34,6)	0.048
Ballon+stenting	30 (66,7)	68 (53,5)	
Ballon therapy	8 (17,8)	15 (11,8)	

* SD: standard deviation,

** n: number of patients,

ASA: acetylsalicylic acid, BNP: brain natriuretic peptid, CABG: coronary arterial by-pass surgery, CRP: C reactive protein, EF: ejection fraction, h: hour, Hb: hemoglobin, HDL: high density lipoprotein, LDL: low density lipoprotein, MI: myocardial infarction, MPV: Mean Platelet Volume, OAD: oral antidiabetics, PLT: platelet, RDW: red cell distribution width, Sedm: Sedimentation, T. Cholesterol: total cholesterol, WBC: white blood cell.

*** Statistic analysis cannot be done due to lack of patient number for clopidogrel under the heading medication usage before MI.

**Table-II:** Effects of variables on the no reflow in univariate analysis and multivariate logistic regression analysis.

Variables	Univariate Analysis		Multivariate Logistic Regression Analysis	
	OR (95% CI)	P Value	OR (95% CI)	P Value
Age	1.81 (0.908 - 3.611)	0.048		
Gander	3.59 (1.626 - 7.983)	0.002		
Diabetes mellitus	2.82 (1.228 - 6.469)	0.023		
Blood glucose level	5.12 (2.128 - 12.347)	<0.001	1.008 (1.002 – 1.013)	0.004
Hemoglobin level	3.43 (1.658 - 7.246)	0.001		
Lymphocyte count	2.59 (1.256 - 5.347)	0.014	0.999 (0.999 – 1.000)	0.026
Mean ST elevation	2.37 (1.310 - 4.978)	0.033		
QT duration	3.18 (1.467 - 6.898)	0.005		
Rescue PCI	4.96 (2.205 - 9.998)	<0.001		
Symptom-ballon time	5.61 (2.678 - 11.755)	<0.001	1.486 (1.248 – 1.770)	<0.001
Ballon+stent therapy	2.77 (1.122 - 6.865)	0.039		

**Fig.1:**
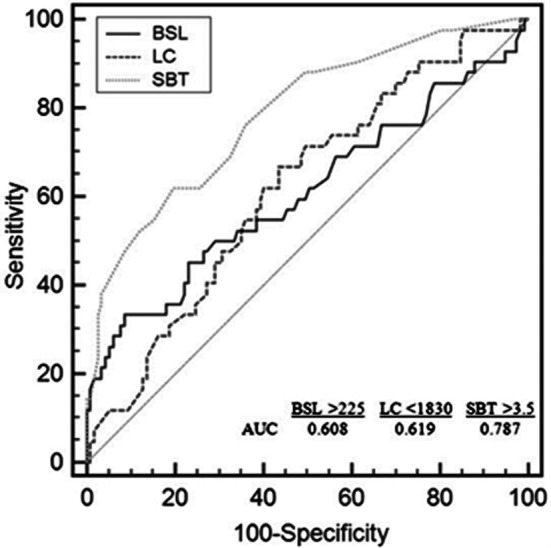
Receiver-operating characteristics curve of blood sugar level (BSL), symptom-balloon time (SBT) & lymphocyte count (LC) for predicting development of no-reflow. (AUC: Area under the Curve).

In ROC analysis, lymphocyte count of <1830 uL at admission was found to be the predictor for the development of no reflow with 54.8% sensitivity and 68.2% specificity(OR = 2.591; 95% CI = 1.256, 5.347; p = 0.014) (Fig.1). The sensitivity was 34.09%, and the specificity was 91.67% in the analysis of ROC curve for the prediction of “no-reflow” in patients with blood glucose level of >225 mg/dL at admission (OR = 5.125; 95% CI = 2.128, 12.347; p <0.001) (Fig.1). In diabetic patients, lower levels of blood glucose were found to increase the risk of “no-reflow”. This value was determined as >143 mg/dL in the analysis of ROC curve in diabetic patients (OR = 2.20; 95% CI = 1.39-3.48; p = 0.047). As the risk of development of “no-reflow” increased with lower blood glucose levels, the contribution of chronic uncontrolled high blood sugar to development of “no-reflow” was evaluated in a separate group in diabetic patients (n = 29). HbA1c level above 7 was used as the criterion to include the patients in the chronic uncontrolled blood sugar group. It was found that HbA1c level of >7 (n = 11) had no influence on the development of “no-reflow” (5 (45%) vs. 6 (54.5%); p = 1.000).

**Fig.2:**
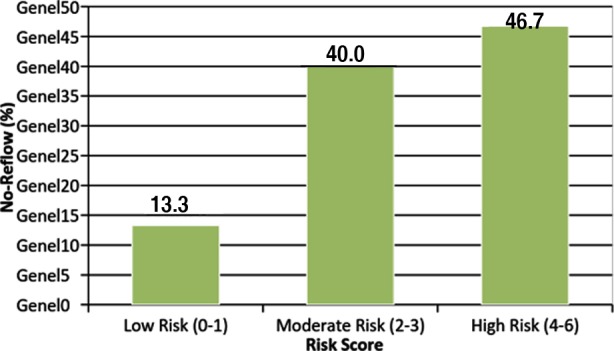
Rates of no-reflow in patients with low, moderate and high risk scores.

The sensitivity was 59.09%, and the specificity was 79.53% in the analysis of ROC curve for the prediction of “no-reflow” in patients with the time of symptom-onset-to-balloon-time >3.5 h (OR = 5.61; 95% CI = 2.678, 11.755; p <0.001) (Fig.2). The mean symptom-onset-to-balloon-time of primary (n = 152, 87.9%) and rescue PCIs (n = 21, 12.1%) were determined as 2.97 ±3.16 h vs. 5.61 ±3.57 h respectively. Rescue PCI was found to increase the risk of the development of no-flow in STEMI patients (p <0.001). In the analysis of ROC curve, it was found that the symptom-onset-to-balloon time of >2 h increased the risk of the development of “no-reflow” in the rescue PCI patients (OR = 4.96; 95% CI = 2.205, 9.998; p <0.001).

**Fig.3:**
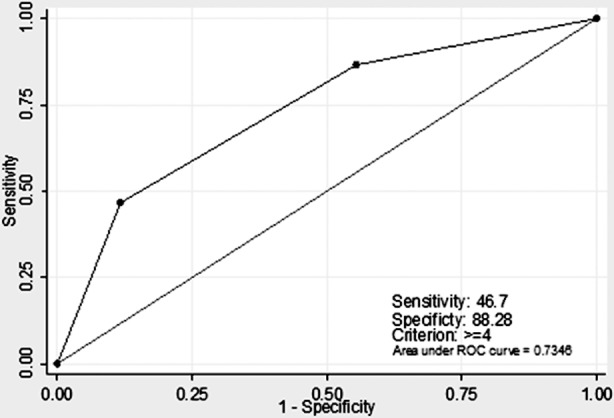
ROC analysis of the no-reflow risk model in the study group.

The independent predictors of “no-reflow” phenomenon were selected for the development of a clinical scoring. To calculate a risk score, we assigned each of the three variables a number of points that were proportional to its area under ROC (AUROC) curves. Table-III shows the variables from the score development set which were included in the final logistic regression model, alongside their associated score component values. The patients were categorized into three groups on the basis of the score as follows: low (risk score: 0-1 (n = 6)), moderate (risk score: 2-3 (n = 18)), and high (risk score: 4-6 (n = 21)). The incidence rates of “no-reflow” in patients with low, moderate, and high risk factors were 13.3%, 40.0%, and 46.7%, respectively. The AUROC was 0.734 (95% CI = 0.654, 0.814; Fig.3), which indicates the ability to efficiently discriminate between patients with various risk levels of the development of “no-reflow” in the study group.

**Table-III:** Clinical risk scores for no-reflow.

Factors	Score value
*Symptom-ballon time*	
≥3,5h	3
<3,5h	0
*Lymphocyte count*	
≥ 1830 uL	2
<1830 uL	0
*Blood sugar level*	
≥225mg/dl	1
<225 mg/dl	0

## DISCUSSION

The parameters that are the independent predictors of “no-reflow” phenomenon are simple laboratory and clinical anamnesis informations. Clinical anamnesis information based on data at presentation, blood sugar level can be achieved by bed sided measurement and blood count is the only acquired laboratory data before the procedure especially in the first 20 minutes of admission to the hospital. With the risk scoring method performed by using the available simple clinical findings, the risk of “no-reflow” can be predicted confidentially before the initiation of PCI treatment, and the prognosis can be improved by decreasing the risk of “no-reflow” with suggested therapies. Other findings of our study were similar with the ones available in literature and validate the importance of the seemingly simple data prior to the procedure. Based on this, the results of the study were interpreted as discussed below:

Hyperglycemia can be seen in the course of acute MI irrelative to DM; and it is associated with increased mortality after MI.^1^ The relationship between “no-reflow” phenomenon and acute hyperglycemia can be explained by a lot of mechanisms. First, there is an increase in the obstruction of capillary bed with leucocytes by increasing the levels of ICAM-1^2^ or P-selectin.^3^ The accumulation of leucocytes in coronary capillary bed after coronary perfusion is higher in diabetic animal hearts when compared to non-diabetic animals.^4^ Leucocyte plugs in capillary bed are among the factors that contribute to the development of “no-reflow” phenomenon. Moreover, hyperglycemia increases the occurrence of thrombus. The occurrence of microthrombus is one of the key reasons of “no-reflow” phenomenon. Finally, it is suggested that hyperglycemia is associated with reperfusion injury. In the heart of mouse, myocardial reperfusion is increased by hyperglycemia that causes the increment of the adhesion of leucocytes to capillary bed and production of free oxygen radicals.^5^ High blood glucose level at reference is associated with high mortality in diabetic patients that are admitted to hospital because of STEMI.^6^,^7^ The regulation of blood sugar with insulin dose during the course of MI in diabetic patients reduced the long term mortality when compared to oral anti-diabetic treatment.^8^,^9^ The normal blood sugar value is suggested to be 90-140 mg/dL.^10^ A cut-off value (143 mg/dL) of blood sugar in diabetic patients which was determined by ROC curve for diabetic patients, is a value to target blood sugar level when identified in diabetic patients undergoing the course of STEMI. In concordance with our current knowledge, the course of blood sugar level higher than the target value increased not only the mortality but also the risk of the development of “no-reflow” in diabetic patients of the study. It can be suggested that the effect of acute high blood sugar on the increase in mortality is related to the increased frequency of “no-reflow” in diabetic patients. The relationship between the indicator of chronic hyperglycemia (HbA1c >7) and the development of “no-reflow” was evaluated in diabetic patients. It was determined that the risk of development of “no-reflow” is higher in diabetic patients irrespective of the HbA1c level. Another data that supports the current findings is the identification of the choice of OAD or else OAD + insulin treatment has no effect on the development of “no-reflow”. According to this, high chronic blood sugar has no effect on the development of “no-reflow” but high acute blood sugar increases the risk. While chronic hyperglycemia is a risk factor for coronary artery disease, acute hyperglycemia is a risk factor for increased mortality in the course of MI.

Leukocyte count increases in the course of acute MI, and this is related with the occurrence of adverse event in the course of acute MI.^11^ Among the white blood cells, while the number of neutrophil and monocyte increases, the number of lymphocyte decreases. It is not clear which leukocyte sub-group is best correlated with adverse events for predicting “no-reflow” phenomenon. Lymphocytes play a prominent role in altering the inflammatory responses in atherosclerotic processes.^12^ Lymphopenia is a condition that occurs due to the increased corticosteroid levels in acute stress conditions^13^, and it is related to mortality after acute MI.^14^ In our study, lymphocyte count is found significantly lower in “no-reflow” developer group, and it is identified as an independent predictor for the development of “no-reflow”. This study indicated that the development of “no-reflow” can be best predicted by decreasing the number of lymphocytes among the white blood cells.

The relationship between symptom-onset-to-balloon-time and “no-reflow” development which is shown in the study is in line with available literature.^15^ Thrombi in early period of MI are rich in thrombocytes, and their treatment is relatively easier with pharmacotherapy. Erythrocyte load increases with prolongation of reperfusion time and thrombi become more resistant. Delayed reperfusion can result in thrombus that has an increased distal embolization risk, and more difficult to ensure TIMI-3 flow, which is more organized and older.^16^ Therefore, the risk of development of “no-reflow” is increased in delayed reperfusion. In concordance with this finding, development of “no-reflow” is more frequent in patients who underwent rescue PCI in the study. “No-reflow” frequency was found increased in over 2 hour’s symptom-onset-to-balloon-time in rescue PCI patients which is a shorter period for all group. Based on this data, it can be suggested that a thrombus which has a structure that cannot be dissolved with fibrinolytic treatment increases the risk of the development of “no-reflow” in a shorter period of time. This data is supported by the study performed by Zalewski et al.^17^ which discusses the association between the specialties of fibrin structure and “no-reflow” development for the first time. The presence of smaller pores in fibrin ball, high level of fibrinogen, and long time of lysis were found related with “no-reflow” development.

### Limitations

There are some limitations in our study. First of all is the design of the study which is single centered, non-randomized, retrospective, and performed in relatively small patient group. Second, evaluated laboratory parameters are not in the same extent for individual patient and some parameters were measured in much smaller groups. Third, for evaluation of reperfusion, grading of TIMI was done instead of myocardial contrast echocardiography.

## CONCLUSION

Ordinary clinic, ECG, and laboratory data offer ancillary information in decreasing long term morbidity and mortality in STEMI patients. Risk scoring method developed from simple clinical data is useful and efficient for the prediction of the risk of “no-reflow” development. The identified risk scoring method can have significant improvement in the prognosis of STEMI patients with administration of medical treatment and/or specific catheters intended to prevent the development of “no-reflow”.
